# Difficult Airway Due to an Undiagnosed Subglottic Tumor: A Case Report

**DOI:** 10.1097/MD.0000000000003383

**Published:** 2016-04-18

**Authors:** Kohji Uzawa, Joho Tokumine, Alan Kawarai Lefor, Toshiyuki Takagi, Kunitaro Watanabe, Tomoko Yorozu

**Affiliations:** From the Department of Anesthesiology (KU, JT, KW, TY), Kyorin University School of Medicine, Sinkawa, Mitaka, Tokyo; Department of Surgery (AKL), Jichi Medical University, Yakushiji, Shimotsuke-shi, Tochigi-ken; and Department of Anesthesia (TT), Higashiyamato Hospital, Nangai, Higashiyamato, Tokyo, Japan.

## Abstract

The “cannot ventilate, cannot intubate” scenario during anesthesia induction can be lethal. We present a patient with an undiagnosed subglottic tumor who developed the “cannot ventilate, cannot intubate” situation after induction of general anesthesia, due to the presence of an undiagnosed subglottic tumor.

A 93-year-old woman was brought to the operating room for repair of a femoral neck fracture. Both ventilation and intubation could not be accomplished, and the patient was awakened without complications after trials of maintaining the airway. In order to reverse muscle relaxation, sugammadex was useful to allow resumption of spontaneous breathing.

A difficult airway can be caused by an undiagnosed subglottic tumor. Subglottic tumors can be misdiagnosed as asthma, because the clinical presentation can be very similar. If cricothyrotomy had been performed based on airway management algorithms, the airway may not have been controlled with a possibly fatal outcome. Ultrasound examination of the trachea may be useful to diagnose obstructive lesions in the airway.

## INTRODUCTION

Intubation of patients in the operating room usually proceeds without difficulty, but anesthesiologists must be prepared to deal with any number of unsuspected conditions that may make intubation difficult or impossible. A deep subglottic tumor may be hard to appreciate on physical examination, but severely limit the ability to intubate a patient. We report a patient with a previously undiagnosed subglottic tumor that made intubation impossible. Patients with subtle signs of respiratory difficulty may benefit from preoperative ultrasound examination of the neck to evaluate the subglottic region.

## CASE PRESENTATION

A 93-year-old woman (height, 140 cm, weight, 38 kg, BMI 19) was brought to the operating room for repair of a femoral neck fracture. Her medical history included hypertension, diabetes mellitus, chronic heart failure, and Alzheimer's disease. Due to her altered mental status, there was no way to obtain an adequate review of systems regarding her respiratory status. She was agitated and had a severely deformed spine. Physical examination showed a slight biphasic wheeze on chest auscultation. Preoperative laboratory evaluation was unremarkable.

General anesthesia was induced with propofol 80 mg and fentanyl 100 μg after preoxygenation with a facemask. Mask ventilation was difficult, oral airway devices were ineffective, and the laryngeal mask failed to ventilate. The patient's SpO_2_ gradually decreased, and tracheal intubation was attempted. The Cormack–Lehane grade was I, but the vocal cords appeared closed. Therefore, rocuronium 15 mg was administered to reattempt intubation. Both the tracheal tube and the tube introducer were difficult to insert. Sugammadex 200 mg was administered as soon as the SpO_2_ decreased to 50%. High-pressure manual ventilation continued until spontaneous respiration returned. At this point, the patient was awake without complications.

A lateral femoral cutaneous nerve block was then performed, and the operation proceeded with placement of an intramedullary nail. In order to assure adequate pain relief during manual reduction of the femoral fracture, local anesthetics were injected as needed.

**FIGURE 1:**
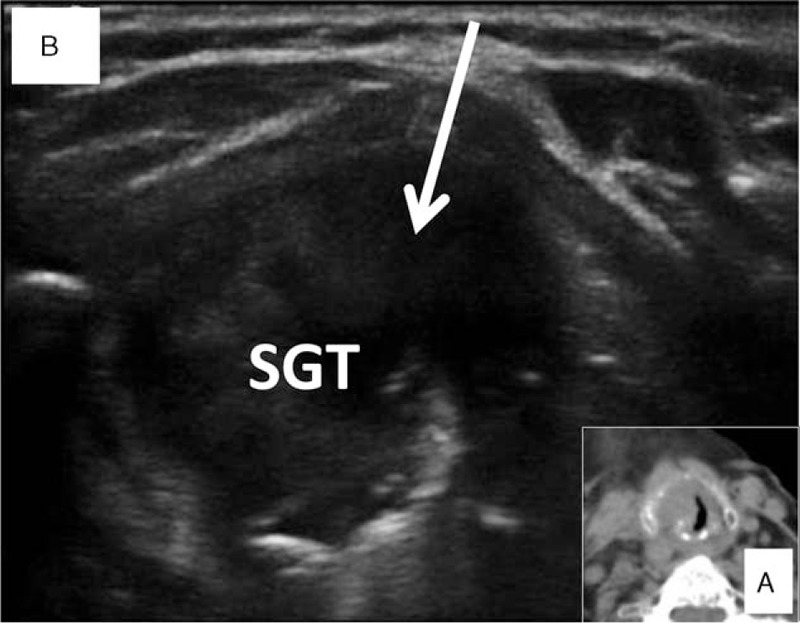
Panel A: Transverse view of the computed tomography scan shows a subglottic mass. Panel B: Ultrasound view at the level of the cricothyroid membrane shows a mass. (SGT = subglottic tumor, arrow indicates the direction of a cricothyrotomy).

Postoperative computed tomography scan showed a subglottic tumor (Figure 1A). Ultrasound examination showed a tumor located just posterior to the cricothyroid membrane (Figure 1B), which explains the difficulty encountered in the operating room during attempted intubation.

## DISCUSSION

Subglottic tumors are rare.^1^ These tumors include a wide variety of lesions such as papillomas, hemangiomas, fibromas, chondromas, myxomas, neurofibroma, epidermoid cancer, chondrosarcoma, and others.^2^ Subglottic tumors can be misdiagnosed as asthma, because some of the symptoms due to a subglottic tumor such as dyspnea, cough, stridor, and biphasic wheeze are similar to the symptoms of asthma.^3–5^

In the care of this patient, we applied the algorithm for difficult airway management^6–8^ and rescued the patient from a “cannot ventilate, cannot intubate” situation. The algorithm also includes performance of a cricothyrotomy. However, cricothyrotomy might have failed and worsened the situation in this particular patient. Emergency percutaneous cricothyrotomy is the procedure of last resort. A meta-analysis of cricothyrotomy showed that needle cricothyrotomy has a low success rate (65.8%) compared to surgical cricothyrotomy (90.5%).^9^ A needle cricothyrotomy can lead to major complications such as perforation of the esophagus or massive bleeding due to arterial injury.^10^ Ultrasound guidance may increase the success rate of percutaneous needle cricothyrotomy,^11,12^ but using it in emergent clinical situations is not always feasible due of technical aspects of using ultrasound.^13^

Preanesthetic evaluation of the patient's neck with a thorough physical examination and ultrasound^13,14^ may be helpful, especially if a difficult airway is suspected. Deep subglottic tumors may be difficult to appreciate despite a complete physical examination due to their location. We recommend adding the risk factor “biphasic wheeze” for a “cannot ventilate cannot intubate” situation. In such patients, ultrasound examination of the trachea behind the cricothyroid membrane should be performed to eliminate a mass as the cause of stridor or other symptoms.
